# Analysis of age-specified and genotype distribution of HPV multiple infections in the Chinese population

**DOI:** 10.1038/s41598-024-53271-1

**Published:** 2024-02-01

**Authors:** Yu-Xia Zhou, Xiao-Hui Ma, Ting-Ting Wang, Xiao-Li Qu, Xiao-Qian Zhang

**Affiliations:** Key Laboratory of Birth Regulation and Control Technology of National Health Commission of China, Maternal and Child Health Care Hospital Affiliated to Qingdao University, 238 Jingshi East Road, Jinan, 250014 Shandong China

**Keywords:** Microbiology, Medical research

## Abstract

Multiple infections are a key component of HPV pathogenesis and have a direct impact on how an infection turns out. It’s crucial to look at the associations between HPV multiple infections and both age and HPV genotypes in the Chinese population, searching for the causative factors of multiple infections with a view to providing new ideas for the treatment and prevention of multiple infections. In this study, we retrospectively analyzed the data of HPV infections among outpatients from the 2019 year to the 2021 year of Shandong Maternal and Child Health Hospital. Analyzed the correlation between HPV multiple infections and age using logistic regression. Differences in the percentage of multiple infections between age groups were compared using the chi-square test. The chi-square test compared the differences in the distribution of 15 common HPV genotypes in mono- versus multiple infections. A two-dimensional matrix presented the frequency of HPV genotype combinations. Logistics regression analysis showed that age was significantly associated with the occurrence of multiple infections, with a dominance ratio OR 1.026 (95% CI 1.02–1.04). Interestingly, the proportion of HPV multiple infections among HPV-positive individuals increases with age in people older than 30 years of age. The chi-square test showed there was a difference in the distribution of HPV genotypes between multiple infections and mono- HPV infection (χ^2^ = 76.4; p = 0.000), a difference in the composition of HPV genotypes for dual versus single infections (χ^2^ = 90.6; p = 0.000) and a difference in HPV genotypes for triple versus single infections (χ^2^ = 56.7; p = 0.000). A 2 × 2 matrix showed that the combination of HPV52/HPV58 (30; 6.4%) was the combination of the highest frequency of infection for dual infections; The HPV52/HPV58 (21; 4.8%) combination was the highest frequency of HPV triple infection combination. HPV multiple infections were positively correlated with age; increasing age was positively correlated with the proportion of HPV multiple infections in the total infected population; the distribution of the 15 common genotypes of HPV differed between multiple infections and single infections; and HPV52:58 was a common type of infection combination in the Shandong population.

## Introduction

Human papillomavirus HPV is of the few viruses on the planet that can be cancerous, causing a range of cancers including cervical, oral, and anal cancers^1^. Studies have found that multiple infections are an important mode of HPV pathogenesis, significantly increasing the severity of infection and the risk of tissue cancer^2^. With the introduction of HPV vaccination, the role of multiple infections in HPV vaccine escape and new vaccine development has received new attention^3^. It is vital to investigate the association between HPV numerous infections and age as well as HPV genotype because the causes of HPV multiple infections are yet unknown.

Age has a significant impact on when to administer the HPV vaccine^4^. HPV prevalence in women is associated with age, but the relationship between multiple infections and age is somewhat more complicated^5^. Particularly the variation of multiple infections as a proportion of HPV-positive individuals by age, which is still unclear, but may provide an important reference for the timing of HPV vaccination.

More than 200 genotypes of HPV viruses have been identified word widely, exhibiting diverse gene sequences and biological characteristics^6^. It is controversial whether there is an interaction between the two genotypes of HPV in people who have been infected multiple times. Depending on whether the HPV types vaccinated against compete with other HPV types or not, any interaction between coexisting HPV types could make current HPV vaccines that offer primarily type-specific protection either more effective or less effective^7^. Hence, multiple infections provide a natural model for the study of viral HPV genotype interactions, and the comparison of the proportion of HPV genotypes in single and multiple infections will provide new perspectives on HPV genotype interactions^8^.

This research uses logistic regression to analyze the correlation between age and multiple infections and uses the chi-square test to compare the changes in the proportion of multiple infections in positive individuals by age, in an attempt to clarify the correlation between age and HPV multiple infections. Comparing the percentage of 15 common HPV genotypes in multiple versus single infections using the chi-square test, and using a matrix to show the combination of HPV genotypes, with the goal of determining whether there is an interaction between HPVs in multiple infections.

## Methods

### Study population

The study population originated from Jinan City, Shandong Province, China, an important transportation hub and capital city in central-eastern China, with a total population of more than 10 million, which is a typical Han Chinese-settled city in northern China. The study population was derived from outpatients of the Department of Gynecology of Shandong Maternal and Child Health Hospital.

### Research ethics

The study is an observational study that does not involve intervention experiments and does not pose a threat to the personal safety of the subjects. The study process was in accordance with the Declaration of Helsinki, approved by the Ethics Committee of Shandong Maternity and Child Health Hospital, and the participants were informed of the study process and the purpose of the study, and informed consent was obtained from the participants.

### Exclusion criteria of the study population

#### Exclusion criteria

Excluding people with underlying diseases, including malignant tumors, cardiovascular diseases, renal diseases, diabetes mellitus and other diseases.

### HPV testing

Cervical aspirates are collected and transported to the laboratory in a specific preservation solution. The HPV genotyping kit (Shanghai, China, Zhijiang Bio) was utilized to perform HPV genotyping by implementing fluorescent quantitative PCR, and the experimental procedures were referred to the steps of HPV genotyping kit. The HPV16, HPV18, HPV31, HPV33, HPV35, HPV39, HPV45, HPV51, HPV52, HPV56, HPV58, HPV59, HPV66, HPV68, and HPV82 genotypes were identified and genotyped using this kit.

### Age grouping

The population included in the study was divided into < 25 years; 26–30 years; 31–35 years; 36–40 years; 41–45 years; 46–50 years; 51–55 years; 56–60 years; and > 61 years groups according to age.

### Statistical analysis

**Figure 1:**
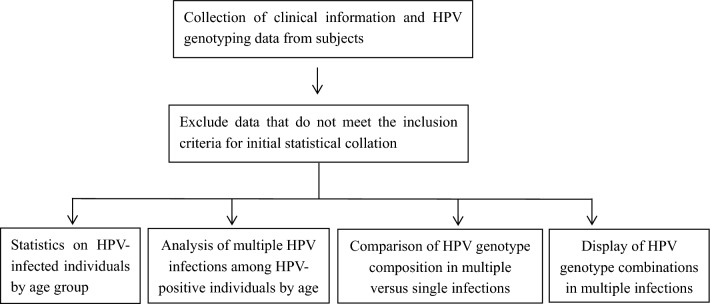
Flow chart for HPV data analysis. Use a flowchart to present the main processes and steps of this study.

Differences in the number of mono- and multiple HPV infections in each age group were analyzed using the chi-square test, with the (31–35) years age group as the reference. Age and multiple HPV correlations were analyzed by chi-square test for ordered categorical variables, and age and the presence or absence of multiple HPV infections were analyzed by logistics regression. The chi-square test was used to test the difference in genotypic composition between HPV single infection and multiple infections. 2 × 2 matrices indicated the frequency of present HPV genotypic combinations. Data were analyzed using SPSS 22.0 software. The flow chart of data processing is presented in Fig. 1. The Fig. 3 were drawing using PPT in office software.

## Results

### Description of the distribution of HPV infection in each age group

The infection rate of HPV multiple infections first increases and then decreases with age. HPV multiple and mono-infections decreased with age in the younger than 30 age group and increased in the older than 30 age group (Table 1). The > 25 age group had the highest rates of dual (11; 8.4%) and triple (12; 9.2%) infections. The 26–30 age group had the lowest dual (101; 2.9%) and triple (40; 2.9%) infection rates. 56–60 age group had higher levels of dual (19; 5.2%) and triple (11; 3.0%) infection rates.

**Table 1:** The age-specific prevalence of the HPV^+^

Age	HPV^+^								
	HPV^+^		Single HPV^+^		Double HPV^+^		Triple HPV^+^		Total
	No.	Ratio (%)	No.	Ratio (%)	No.	Ratio (%)	No.	Ratio (%)	No.
≤ 20	53	40.5	30	22.9	11	8.4	12	9.2	131
21–25	379	26.0	253	17.3	89	6.1	37	2.5	3824
26–30	608	17.6	467	13.5	101	2.9	40	1.2	3463
31–35	724	18.9	554	14.5	123	3.2	47	1.2	1461
36–40	396	19.4	306	15.0	70	3.4	20	1.0	2039
41–45	218	20.8	172	16.4	34	3.2	12	1.1	1050
46–50	148	20.6	113	15.7	26	3.6	9	1.3	719
51–55	135	22.5	103	17.2	24	4.0	8	1.3	599
56–60	88	24.2	58	16.0	19	5.2	11	3.0	363
> 60	67	25.7	36	13.8	21	8.1	10	3.8	261

The age-specific prevalence are calculated by (the total HPV positive, the single HPV positive individual, double HPV positive individual or triple HPV positive individual) divided (total age-specific people) respectively.

### Logistics regression analysis of the correlation between the occurrence of HPV multiple infections and age

Logistics regression analysis of the correlation between age and multiple HPV in people > 30 years of age showed data in Table 2 that age was significantly associated with the occurrence of multiple infections (p = 0.000), with a dominance ratio OR 1.026 (95% CI 1.02–1.04). Also, logistics regression analysis found that age was significantly associated with HPV infection with a superiority ratio OR 1.013 (95% CI 1.01–1.02).

**Table 2:** Linear regression analysis of age on HPV infection.

HPV infection	Wald	p	OR	95% CI
HPV+	18.3	0.000	1.013	1.01–1.02
Multiples HPV+	22.3	0.000	1.026	1.02–1.04

Selected the individuals more than 30-year-old.

### Comparison of HPV multiple infections with single infections by age group

**Figure 2:**
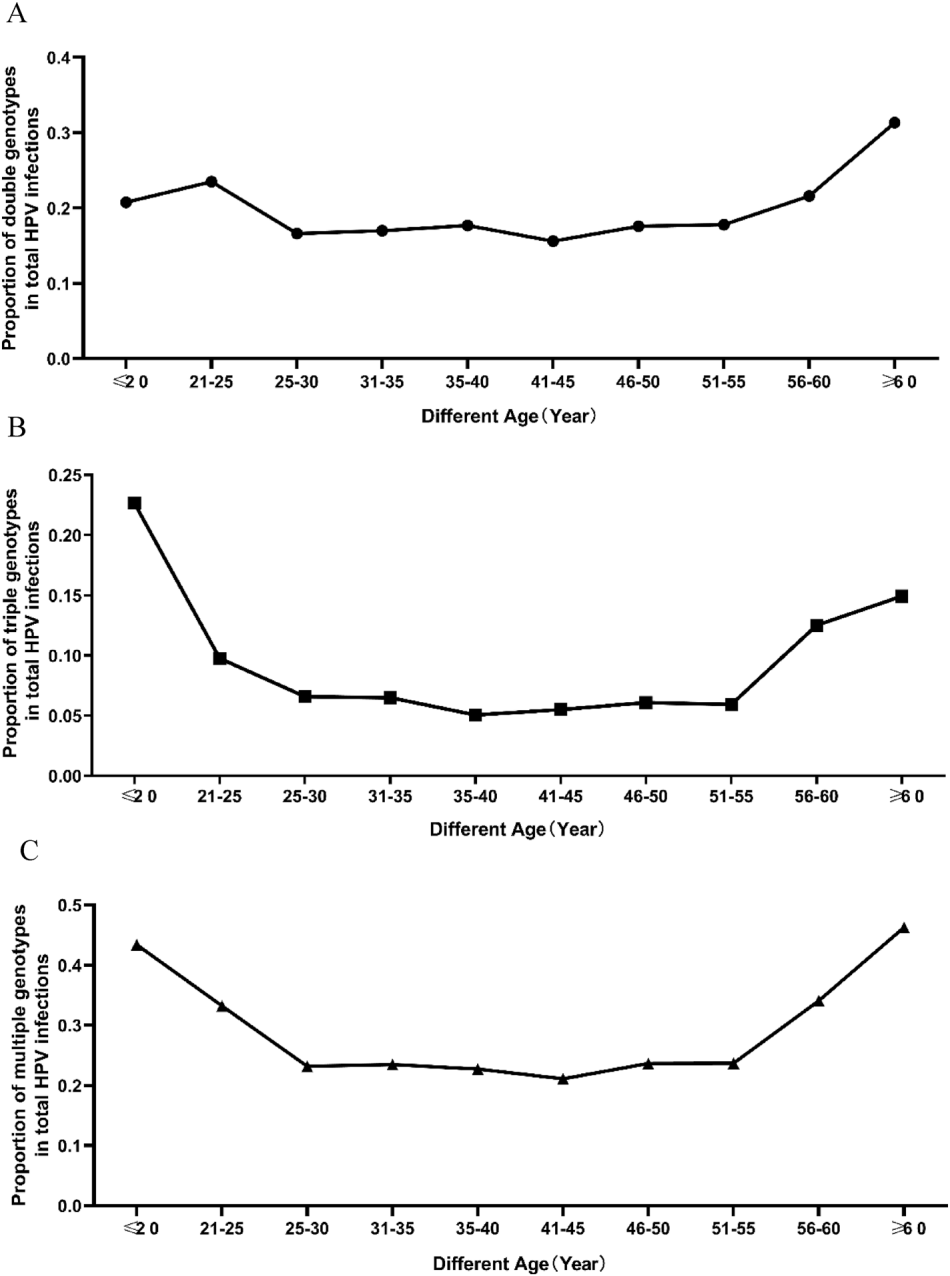
Percentage of multiple HPV-infected persons among the number of HPV-positive individuals. (**A**) Percentage of double HPV-infected persons among the number of HPV-positive individuals. (**B**) Percentage of triple HPV infected persons among the number of HPV-positive individuals. (**C**) Percentage of multiple HPV-infected persons among the number of HPV-positive individuals. Percentage was calculated by multiplied individuals divided by HPV-positive individuals.

The chi-square test was used to determine if the prevalence of HPV multiple infections and HPV single infections different by age group. HPV-infected individuals were used as the sample size, Chi-square test showed that there was a significant difference between HPV multiple infections and HPV single infections in the group of 21–25 years compared to 31–35 years (χ^2^ = 12.1; p = 0.001; Table 3); and there was a significant difference between HPV multiple infections and HPV single infections in the group of 56–60 years compared to 31–35 years (χ^2^ = 4.8; p = 0.029; Table 3). The ratio of HPV multiple infections to HPV-positive individuals in each age group was calculated, and the pattern of change of the ratio in each age group was shown in a line graph. Figure 2 shows that the ratio of multiple infections decreased with age at age < 30 years, the ratio of multiple infections was basically flat between 30 and 55 years, and the ratio of multiple infections increased with age after > 55 years.

**Table 3:** Chi-square test for difference age groups among HPV^+^ individuals.

Age	HPV^+^					
	Multiples HPV^+^		Double HPV^+^		Triple HPV^+^	
	χ^2^	p	χ^2^	p	χ^2^	p
≤ 20	10.5	**0.001**	0.5	0.484	18.4	**0.000**
21–25	12.1	**0.001**	6.8	**0.009**	3.8	0.052
26–30	0.1	0.901	0.1	0.855	0.1	0.949
36–40	0.1	0.775	0.1	0.771	0.9	0.331
41–45	0.5	0.464	0.2	0.629	0.3	0.598
46–50	0.1	0.965	0.1	0.865	0.1	0.853
51–55	0.1	0.955	0.1	0.823	0.1	0.805
56–60	4.8	**0.029**	1.2	0.283	4.3	**0.039**
> 60	16.8	**0.000**	8.5	**0.004**	6.5	**0.011**

All groups were compared with the (31–35) age group.

Significant values are in bold.

### Comparison of the differences in HPV genotype distribution between the multiple infection group and the single infection

The chi-square test was used to compare the distribution of HPV genotypes in the multiple-infection group with that in the single infection group, using HPV-infected individuals as the sample size. The results showed that there was a difference in the distribution of HPV genotypes between multiple infection and single infection (χ^2^ = 76.4; p = 0.000; Table 4), a difference in the distribution of HPV genotypes between double infection and single infection (χ^2^ = 90.6; p = 0.000; Table 4), and a difference in the composition of HPV genotypes between triple infection and mono- HPV infection (χ^2^ = 56.7; p = 0.000; Table 4).

**Table 4:** Chi-square test for genotype differences between single and multiple HPV infections.

Chi-square	Genotypes		
	Multiple HPV^+^	Double HPV^+^	Triple HPV^+^
χ^2^	76.4	90.6	56.7
p	0.000	0.000	0.000

Single and multiple infections contain 15 genotypes.

### Description of HPV genotype combinations in HPV multiple infections

HPV genotype combinations in dual infections were demonstrated by a 2 × 2 matrix (Table 5). The results showed that HPV52:58 (30; 6.4%) was the most prevalent combination type, followed by HPV52:16 (27; 5.8%), and then HPV58:16 (23; 4.9%). HPV genotype combinations in triple infections were demonstrated by 2 × 2 matrix (Table 6). According to the results, the most common HPV genotype combination type was HPV52:58 (21; 4.8%), followed by HPV52:16 (20; 4.5%), and then HPV59:52 (17; 3.9%).

**Table 5:** The different HPV genotype combinations among dual HPV infections.

Type	Type													
	18	31	33	35	39	45	51	52	56	58	59	66	68	82
16	5	4	8	4	14	3	10	27	8	23	8	7	6	3
18		1	4	2	6	3	6	6	5	2	1	1	1	1
31			2	1	3	2	3	6	4	6	0	3	3	1
33				0	4	1	2	7	5	1	1	0	2	0
35					1	0	1	3	1	6	0	4	4	0
39						2	4	13	3	10	2	1	2	2
45							0	1	0	1	0	0	1	8
51								21	3	8	5	5	4	3
52									7	30	5	15	11	3
56										6	2	12	3	1
58											2	5	6	2
59												0	6	1
66													4	1
68														0

**Table 6:** The different HPV genotype combinations among triple HPV infection.

Type	Type													
	18	31	33	35	39	45	51	52	56	58	59	66	68	82
16	5	2	5	4	5	0	4	20	4	13	11	8	7	2
18		5	1	1	3	1	4	4	0	5	3	3	1	0
31			2	2	2	0	4	8	1	6	2	4	2	0
33				1	1	1	3	5	4	2	2	4	5	0
35					2	1	2	9	2	7	2	4	1	2
39						1	4	13	4	5	6	4	6	0
45							1	1	2	2	2	0	0	2
51								12	7	7	7	3	4	2
52									8	21	17	10	13	3
56										3	0	10	5	0
58											12	8	7	0
59												2	5	1
66													2	0
68														2

## Discussion

### Incidence of multiple HPV infections associated with age

There were differences in the prevalence of multiple HPV infections by age, and logistics regression analysis revealed a significant positive correlation between the occurrence of multiple HPV infections and age, with a correlation coefficient of only 1.02, but the correlation was significant, which is generally consistent with studies of HPV prevalence in other geographic areas of the Chinese population^9,10^. For the first time, this research focus on the account of increased multiple infections in the total number of infections, which can provide new perspectives on the factors influencing multiple infections. Interesting, the percentage of multiple infections increases steadily with age (more than 40), raising from 21.1% in the 41–45 group to 46.3% in the > 60 years old group. These implying age-related female hormonal changes affect the immune status may be an important reason for this phenomenon. More thoughtfully, the cumulative effect of HPV infection may be an irreplaceable driver of this phenomenon^11^. The cumulative effect of HPV infection simply means that the persistent HPV infection disrupts host immunity and promotes secondary HPV infections, as discussed in more detail below (4 paragraph of the “Discussion” section)^12^. There is limitation in this study, the small number of people below 25 and above 60 years of age who met the criteria for inclusion may affect the accuracy of the results.

### Multiple types of infections are observed more frequently than predicted frequencies

Considering the vast natural population, which is much larger than the sample of this present study, it can be roughly assumed that this study fulfills the following conditions: a. The rate of HPV infection in this program is approximately the population prevalence rate; b. The occurrence of HPV infection is an independent stochastic event. According to the mathematical probability calculation formula, the prevalence of HPV52 present as “a”, which can be found from Table 1; the prevalence of HPV16 present as “b” which also can be found from Table 1; the prevalence of HPV52:16 combination present as “p” Based on the basic probability formula p = a × b, predict the probable incidence of HPV52:16:

- a = 0.047 (Table 1);
- b = 0.035 (Table 1);
- p = 0.047 × 0.035 = 0.0017.

The predicted HPV52:16 combination probability is 0.0017, which is smaller than statistical HPV52:16 combination probability 0.0037. The actual probability of having a double infection is greater than the probability of having two separate infections. Similar findings have been reported in many countries around the world, including the United States, India, Mexico, Kenya, and others countries, it is remain controversial to how to explain the observed higher than predicted incidence of HPV multiple infections^13–15^?

**Figure 3:**
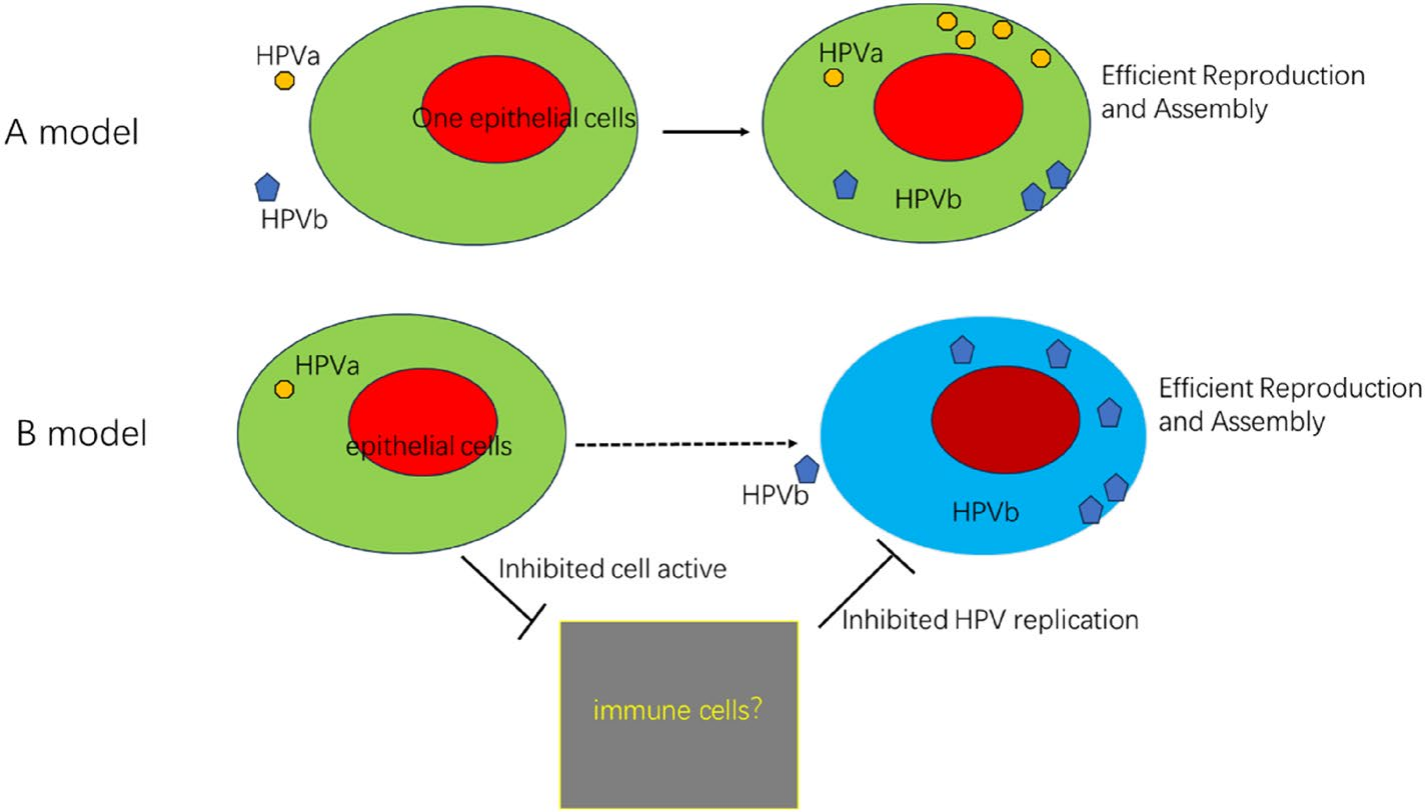
Schematic diagram of two models for explaining interactions between HPVs. (**A**) The HPV co-infection cell model. The two viruses efficiently infiltrate and assemble inside the same cervical epithelial cell through a process known as synergistic regulation. (**B**) The HPV induced immune-suppressive microenvironment. HPV viral infiltration decreases resistance to secondary infection, suppresses host immunity by inhibiting immune signaling activation, and promotes secondary viral infection.

A possible mechanism for the high predictive value of multiple HPV gene infections is the synergistic interaction between HPVs during multiple infections, for which we propose two explanatory models (Fig. 3): model of the HPV co-infection in a single cell, and model of the HPV induced immune-suppressive microenvironment^16^. The HPV co-infection in a single cell refers to the two viruses imbibe the same cervical epithelial cell, and are synergistically regulated in a single cell to efficiently complete the infiltration and assembly. Unfortunately, there is no evidence of synergistic replication and assembly of the two viruses in a single cell up to now^17^. Agawa et al., 1993, in plantar warts, found that the same nucleus was infiltrated by both hpv1 and hpv63 in a single cell, but only one virus, hpv63, was expressed and assembled in large numbers^18^. Christensen et al., showed that human foreskin mucosal cells were simultaneously infected with multiple viral components, including Hpv11, HPV40, each HPV type predominantly maintains regional separation within the same papilloma^19^. The HPV infiltration inhibition host model refers to HPV viral infiltration, which inhibits host immunity, reduces resistance to secondary infection, and facilitates secondary viral infection^20^. This is similar to the mechanism of mixed HIV and HPV infections, where HIV impairs host immunity, causing more severe HPV infections^21^. HPV16, 18, and 31 have been found to inhibit the expression of interferon (IFN-κ), a type I IFN constitutively expressed in keratinocytes^22^. Those molecules drive basal expression levels of IFN-stimulated genes (ISGs) and causing the activation of STAT1 and IFIT1, inhibiting HPV virus assembly and replication^23^. In conclusion, primary HPV infection reduces ISG expression, and suppresses the host cervical epithelial innate immune system, favoring the invasion of other types of HPV^24^.

### Differences of HPV genotype distribution between multiple infections and single infections

The main HPV genotypes infecting multiple and single infections were in order: HPV52, HPV16 and HPV58, which was basically consistent with studies in other regions of China^25^. However, this result is different from other regions of the world. HPV-16 and HPV-53 were the most prevalent high-risk HPV genotypes in Lebanon; the predominant HPV genotypes in Iran were HPV6, HPV16, HPV31; in Asuncion, HPV-58, HPV-16 and HPV-51were the most commonly detected HPVs^26–28^. The slightly different findings in this study may be related to the population and geographical distribution. Interesting, the HPV genotypes of multiple versus single infections are identical in all areas investigated. This study is the first to compare the proportion of 15 common HPVs in multiple infections versus single infections, and found differences in their composition, suggesting that HPV genotypes affect single and multiple infection events differently.

This research demonstrated that the most common combination type in double infection and triple infections are HPV52:58, followed by HPV52:16 and again HPV59:52. There are few studies on the types of HPV genotype infection combinations, and this study will provide new ideas for exploring the causative factors of HPV infection combinations and the development of multivalent HPV vaccines.

## Conclusion

In conclusion, by studying multiple HPV infections in a Chinese population, we inferred that HPV multiple infections were positively associated with age; increasing age was positively associated with the proportion of HPV multiple infections in the total infected population; the distribution of the 15 common genotypes of HPV differed between multiple and mono-infected HPVs; and HPV 52:58 was a common type of combination of infections in the Shandong population.

### Disadvantages and shortcomings

The enrolled population was derived from outpatients and not the natural population, first, awareness of HPV screening among individuals in different age groups was an important factor influencing the results of the statistical analysis. Hence, predictions of HPV prevalence and probability of multiple infections may be biased.

The sample size included in this study was relatively limited, especially the amount of multiple HPV infections, and increasing the sample size could significantly increase the reliability of the study.

## Data Availability

All data generated or analyzed during this study are included in this published article.

## Abbreviation

HPV: Human papillomavirus
